# A Statistical Growth Property of Plant Root Architectures

**DOI:** 10.34133/2020/2073723

**Published:** 2020-11-08

**Authors:** Sam Sultan, Joseph Snider, Adam Conn, Mao Li, Christopher N. Topp, Saket Navlakha

**Affiliations:** ^1^Cold Spring Harbor Laboratory, Simons Center for Quantitative Biology, Cold Spring Harbor, NY, USA; ^2^University of California San Diego, Institute for Neural Computation, La Jolla, CA, USA; ^3^Donald Danforth Plant Science Center, St. Louis, MO, USA

## Abstract

Numerous types of biological branching networks, with varying shapes and sizes, are used to acquire and distribute resources. Here, we show that plant root and shoot architectures share a fundamental design property. We studied the spatial density function of plant architectures, which specifies the probability of finding a branch at each location in the 3-dimensional volume occupied by the plant. We analyzed 1645 root architectures from four species and discovered that the spatial density functions of all architectures are population-similar. This means that despite their apparent visual diversity, all of the roots studied share the same basic shape, aside from stretching and compression along orthogonal directions. Moreover, the spatial density of all architectures can be described as variations on a single underlying function: a Gaussian density truncated at a boundary of roughly three standard deviations. Thus, the root density of any architecture requires only four parameters to specify: the total mass of the architecture and the standard deviations of the Gaussian in the three (*x*, *y*, *z*) growth directions. Plant shoot architectures also follow this design form, suggesting that two basic plant transport systems may use similar growth strategies.

## 1. Introduction

Understanding how biological branching networks grow and distribute branches in space has long fascinated mathematical biologists [1]. These networks serve as the basis for acquiring and distributing resources, for example, neural arbors that process information [2–4], vascular networks that circulate blood [5, 6], and plant architectures that transport sugars and nutrients [7–9]. Due to basic similarities in their design and function [10, 11], it is natural to ask whether there are quantitative features shared by several of these networks [8, 12].

Many structural features of plant root architectures have been studied, including root length and root depth [13], the distribution of root hairs [14–16], the size and number of lateral branches [17–20], and biomass allocation to fine roots [21]. More global properties of root shapes have also been analyzed [22], including allometric and fractal scaling [23–25], root foraging precision [26, 27], and topological morphology via persistent homology methods [28]. These properties can affect numerous biological functions performed by root architectures, including anchorage, carbon sequestration, and search for water and nutrients in the soil [29–33]. Identifying the molecular mechanisms that drive the formation of different shapes can aid in uncovering genotype-to-phenotype relationships [34] and in breeding specific traits of interest in crops [35–39].

We focus here on branch density [40, 41] and the spatial distribution of roots [42–44], which have numerous functional implications. Water and nutrients in the soil are ephemeral, distributed heterogeneously within a complex chemical and physical environment and intensely competed for by other organisms. Thus, when and where a plant grows its roots have strong implications for its success in resource capture and therefore its viability. In this foraging process, a plant's limited carbon resources are continually partitioned between the growth of existing roots (elongation) and the generation of new roots (branching), which is conditioned by genetic mechanisms and both internal and external signals. The outcome of these iterative processes at any point in time is reflected by the global properties of the root system architecture, which is a complex 3D shape intimately tied to root system function.

Spatial distributions of root length, biomass, and density reflect the topological and geometric properties of root system architecture [45] and have been used extensively as quantitative summaries to compare the genetic, environmental, and genetic x environmental differences in how roots forage. Significant understanding of root system architecture has developed around mathematical models that fit observations of root systems to spatial density functions, beginning with the simple linear model of Gerwitz and Page [46]. However, these models do not fit all data well; in part, because observations from the field have almost exclusively consisted of sparse, lower dimensional representations of much more complex 3D root structures. Classic and still widely-used methods include analysis of root fragments from slabs taken from trench or monolith excavations, or from soil cores or minirhizotron data [47]. Efforts to better approximate the true root structure from these limited samples have used angular distributions [48] or 3D structural simulations [49] to produce better quantitative relationships between the observable arrangement of roots as they intersect the 2D plane of a slab, to the actual density distribution in 3D space. Current architectural (reviewed by Dunbabin et al. [50]) and density continuum models (reviewed by Dupuy et al. [51]) have been greatly aided by continued improvements in root phenotyping and computer simulation, including the addition of soil chemical and physical parameters that shape outcomes of root structure and resource foraging functions [52, 53]. Furthermore, describing root systems in terms of spatial densities allow a direct comparison to shoot systems.

### 1.1. Our Contributions

Here, we use tools from probability theory to study the spatial density function of root architectures, which describes the probability of finding a branch at each point in the 3D volumetric space occupied by the root system. Our goal is to uncover statistical properties of this function, to study how this function varies across 4 species comprising 10 genotypes, and to determine if there are features of this function shared by all architectures studied. Specifically, we analyzed 1645 3D root architectures from four species (rice, corn, tomato, and *Arabidopsis*), including several genotypes per species. Using statistical moments to characterize the spatial density function of each architecture, we found that all functions are population-similar, i.e., all 1645 architectures analyzed had the same underlying shape and thus could be superimposed on top of each other, modulo compression and stretching along each direction. We then derived an analytical form of the spatial density function and discovered that all root architectures can be closely approximated by a 3D Gaussian function truncated at approximately 3.3 standard deviations. This means that, for each architecture, root density is highest at the center of mass and then decays like a Gaussian function until the boundary of the root system is reached at 3.3 standard deviations from the center. The truncation corresponds to the boundary of the plant, outside of which there is zero density, since plants have a finite spatial domain. This result also implies that the root density of any architecture requires only four parameters to specify: the total mass of the architecture and the standard deviations of the Gaussian in the three growth directions. We also find species-specific variation in this property, which suggests functional specialization across genotypes despite following the same general design template. Finally, prior work found that a Gaussian function truncated at 2.0 standard deviations closely approximated the spatial density function of hundreds of 3D shoot architectures [54]. To our knowledge, this highlights one of the first shared structural features of plant architectures above and below ground.

## 2. Results

### 2.1. Dataset of 3-Dimensional Root System Architectures

Phenotyping root system architectures in their natural habitat remain technologically challenging due to soil and particulates that obfuscate visibility. To overcome these physical barriers, several phenotyping methods have been proposed, each of which employs a different trade-off in terms of accuracy, coverage, throughput, and cost [18, 34]. These methods are based, for example, on ground-penetrating radar [55–57], electric resistivity tomography [56, 58, 59], minirhizotrons [60–64], shovelomics [65, 66], soil coring [67], X-ray tomography [68–71], magnetic resonance imaging [42, 72, 73], and 3D laser scanning [74, 75].

**Figure 1:**
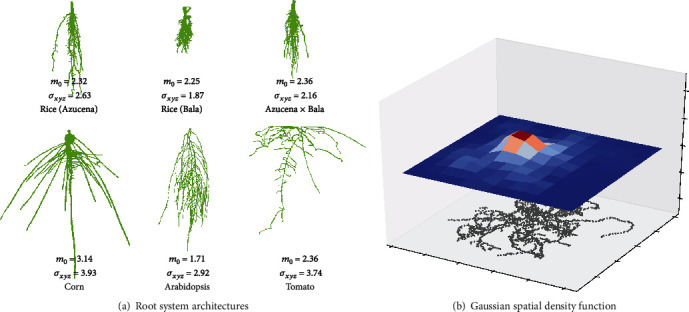
Point clouds of root system architectures and example Gaussian spatial density function. (a) Example architecture point clouds for three rice species (Azucena, Bala, and an F6 recombinant inbred), corn, *Arabidopsis*, and tomato. The moments (*m*_0_ and the size, *σ*_*xyz*_) are shown in log_10_ format. (b) The gray dots show a 2-dimensional projection of a rice (Bala) architecture with its empirical spatial density function above, exemplifying a truncated Gaussian density function. The truncation corresponds to the boundary of the root system in each direction *x*, *y*, and *z*; outside this boundary, there is zero density. Root density is highest at the center of mass (red square) and then decays as you move outward.

Here, we used a controlled environment, gel-based optical imaging platform that allows the 3D architectures of freely-growing root systems to be measured noninvasively in large numbers [35, 75–78] (Supplementary Methods (available here)). This method outputs a point-cloud representation of an architecture (Figure 1(a)). Overall, we collected 1645 architectures from four species, with multiple genotypes per species.

1. Rice [35]. We collected two genotypes of *Oryza sativa* (Bala and Azucena), as well as a third group representing the F6 recombinant inbred lines of the parental cross Azucena x Bala with 171 different families. The recombinant inbred lines provide additional genetic and phenotypic variation within the rice population. In total, there were 36, 29, and 421 individual Azucena, Bala, and Azucena x Bala plants, respectively. Most individual plants were imaged roughly three times on days 12, 14, and 16, respectively, totaling 1358 root architectures.
2. Corn. We collected four genotypes of maize (*Zea mays* L. ssp. *mays*): B73, Mo17, IHP, and ILP. In total, we analyzed 106 architectures, each captured on growth day 6.
3. *Arabidopsis thaliana* [79]. We collected two genotypes: Col-0 and tob1-1 (containing a mutation in an auxin biosynthesis pathway gene). In total, we analyzed 133 architectures representing 31 individual plants, each imaged on approximately days 9, 11, 13, 15, 17, 21, and 25.
4. Tomato. We collected two genotypes of *Solanum lycopersicum*: wildtype cultivar “Moneymaker” and an RNAi line in a nitrogen metabolism gene [80]. In total, we analyzed 48 architectures representing 19 individual plants, each imaged on approximately days 6, 8, 10, 12, and 14.

The plant species chosen represent two major forms of root systems—tap-rooted (tomato, Arabidopsis) and fibrous-rooted (corn, rice)—with fundamentally different developmental patterning. Further, three of the species are important agriculturally (tomato, rice, corn), and one is a traditional model system (*Arabidopsis*). Thus, these species are phylogenetically and developmentally diverse and relevant economically and scientifically. Moreover, the dataset contains multiple genotypes per species and multiple time-points per plant, and it contains architectures ranging over two orders of magnitude in size, from 2,011 to 317,933 cloud points. Thus, this dataset represents a strong benchmark for testing whether a statistical property is shared by a broad class of root architectures.

### 2.2. Describing Root Architectures Using Statistical Moments

To characterize how roots are distributed in space, we studied the root system's spatial density function (Figure 1(b)). This function describes the density of points in the 3D volumetric territory occupied by the roots. The methods used to study the spatial density function are briefly summarized below; full technical details can be found in the Supplement and in prior published reports [54, 81].

Finding properties of the spatial density function that can be compared across many diverse architectures is a formidable challenge due to noise and variability in architecture sizes. For example, grid-counting techniques, which define density as the total sum of points in each voxel, can be highly sensitive to noise and other shifts in form (e.g., rotations and translations). Further, using a small size for each voxel will generate density functions that are sparse and difficult to compare, whereas large-sized voxels may not provide sufficient spatial resolution to make detailed comparisons.

To overcome these challenges, we define the spatial density function by its statistical moments [81]. Knowing all of the moments of a distribution is equivalent to knowing the function that generates the distribution, and the accuracy of the description increases with more moments calculated [82]. Lower order moments capture coarse features of the architecture—e.g., the 0th moment is the total mass of the root system, the 1st moment divided by the total mass is the center of mass, and the 2nd moment is the rotational inertia—whereas higher order moments capture more intricate details, including the shape of individual branches. Further, using moments to study the spatial density function avoids reliance on template matching or other assumptions about the number of functional forms for architectures [83, 84].

To calculate moments, we start with a 3D point cloud of the architecture (Figure 1(a)). A point cloud is a set of *n* points in a Cartesian coordinate system, where each point *p*_*i*_ = (*x*_*i*_, *y*_*i*_, *z*_*i*_) is the 3D position of the point on the surface or interior of the object (root system). Following standard probability theory, the *k*th product moment is defined as:

(1) $$\begin{array}{c} m_{k}=\overset{n}{\underset{i=1}{\sum }}{\left(x_{i}-\overset{¯}{x}\right)}^{k}{\left(y_{i}-\overset{¯}{y}\right)}^{k}{\left(z_{i}-\overset{¯}{z}\right)}^{k}\Delta^{3}, \end{array}$$

where $\overset{¯}{x}$ denotes the center of mass of the root system in the *x*-direction (i.e., the mean of the *x*-coordinates of each point), and Δ^3^ is the volume of a voxel in mm^3^. The resolution of the camera system and the resulting density of points varied from root to root depending on the experimental setup. We calculate moments for even values of *k* (to ensure all moments are positive) and thus leave out the absolute value sign. Following the definition, the 0th moment is equal to the total mass —*m*_0_ = ∑_*i*=1_^*n*^Δ^3^— which is used to normalize for size.

### 2.3. Are Root System Architectures Population-Similar?

We first describe the goal of our analysis intuitively, and then, we formalize these notions mathematically.

Our first goal is to use architecture moments to test if all spatial density functions are population-similar. This asks: do all density functions have the same shape, modulo stretching and compression of the architecture along one or more directions? Whether two root systems share the same density function can be difficult to assess visually. For example, two root systems may have the same shape, but one may be stretched along the *y* (up-down) direction to forage deeper in search for water, whereas another root system may develop lateral roots that stretch along the *x* direction. Similarly, two architectures scaled to be the same size could superimpose exactly, but this may be difficult to tell when comparing a large versus a small root system.

Population similarity is different than the commonly studied property of self-similarity [1, 8, 84]. “Self-similarity” is often associated with having the property that a single structure looks similar at all magnification scales (e.g., it is fractal). Population-similarity considers not a single structure, but rather a population of structures, and asks whether all of them can be viewed as variations, via stretching or compression, of a single form [81]. In other words, different root spatial density functions are population-similar if they can be “transformed” into one another by expanding or contracting root density along orthogonal spatial dimensions [54].

The concept of equivalence across stretching translates mathematically in terms of a density function *f*(**x**; *λ*) that measures the probability that an object of size *λ* has mass at 3D position **x**. The density function depends explicitly on spatial location **x** and the overall scale, *λ*, of the architecture. Without loss of generality, we conventionally define spatial location for each architecture with respect to its center of mass. However, we do not know the length scale, *λ*, of each architecture because, unless we are generating the architectures ourselves, we only measure the consequences of changing *λ*, not *λ* itself.

To rewrite everything in terms of measurable quantities, consider the mathematical definition of equivalence across stretching:

(2) $$\begin{array}{c} f\left(x;\lambda \right)=c\lambda^{b}f_{R}\left(x/\lambda ;1\right). \end{array}$$

This formulation expresses all possible architectures in terms of a single reference architecture, *f*_*R*_, that is stretched (divided) by a factor of *λ* and scaled (multiplied) by a power law, *λ*^*b*^. The key advantage of this formulation is the separation between how the architectures change size and shape, reflected in the power law and *f*_*R*_ terms, respectively. As detailed above, the density function itself is experimentally inaccessible, but we can measure its moments from the point cloud data using Eqn. (1). Moments of the theoretical density function may also be calculated as

(3) $$\begin{array}{c} \log\left[\frac{m_{k}}{m_{0}}\right]\sim k\log\left[\sigma_{xyz}\right]+c, \end{array}$$

where the information about population similarity is encoded in the first term, and the form of the reference architecture is encoded in the single parameter, *c*. See Supplementary Information for further derivations of the population similarity test and power-law scaling.

**Figure 2:**
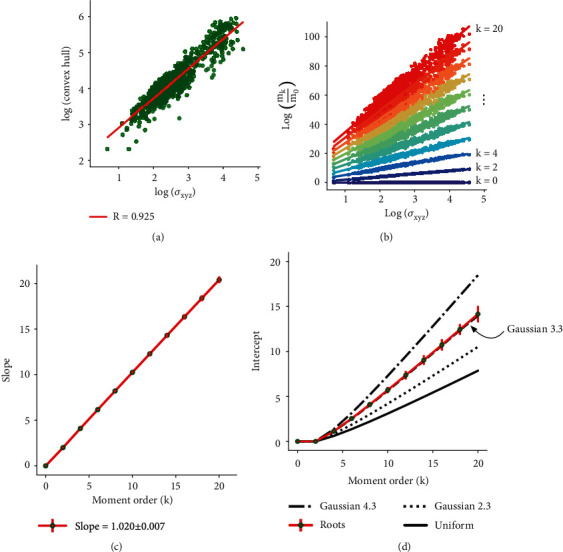
Root architectures are population-similar and have Gaussian spatial densities. (a) Log-log plot showing that a moments-derived measure of architecture size (standard deviation, *x*-axis) is highly correlated with a common measure of size (convex hull volume, *y*-axis). (b) Step one of the population-similarity test, plotting the log of *m*_*k*_/*m*_0_ (*y*-axis) versus the log of architecture size (*x*-axis) for all 1645 architectures. Only even moment orders between 0 and 20 are plotted. Each root architecture has one dot for each moment order. Straight lines are the least-squares fit for each moment order. (c) Step two of the population-similarity test, plotting the moment order (*x*-axis) versus the slope of the corresponding line from panel (b) (*y*-axis). For each moment order, error bars correspond to 99% confidence intervals for each corresponding regression line in panel (b). Error in the legend shows the standard error, computed using bootstrapping. (d) Plots of the intercepts of the lines in panel (b) versus moment order. The intercepts of the data (roots) closely overlaps with the intercepts for a Gaussian spatial density function truncated at 3.3 standard deviations. Intercepts for the uniform and truncated Gaussian spatial densities were computed analytically [81].

Eqn. (3) forms the basis to determine the degree to which root spatial density functions are population similar. This involves two steps:

1. The first step is to plot, log(*m*_*k*_/*m*_0_) versus log(*σ*_*xyz*_) for various values of *k* and for each architecture. If, for each value of *k*, the two have a linear relationship (with a different slope for each *k*), then the architectures share the same function. The term *σ*_*xyz*_ represents a typical measure of size that is computable using only the moments themselves, and no other quantity (Supplementary Methods). This measure is also proportional to another common measure of size, the convex hull volume, i.e., the smallest convex polytope that encloses all the cloud points (Figure 2(a)).
2. The second step is to plot the slope of the lines generated in the first step versus *k*, the moment order. The difference between the slope of this line and 1 denotes the degree to which the architectures are all population similar [81].

Even if roots are population-similar, they may be generated by different population-similar functions, such as a uniform, an exponential, or a Gaussian spatial density. Each species or genotype may also belong to its own functional class; e.g., tomato architectures may have a uniform spatial density, whereas corn architectures may have a Gaussian density. This test can determine how many different classes of population-similar functions are required to describe the root architectures because architectures from one class will fall on one line, and architectures for another class will fall on a different line, for each value of *k*. Thus, this test quantifies the degree to which architectures are population-similar and the number of functional classes of architectures.

For the first test, we plotted log(*m*_*k*_/*m*_0_) versus log(*σ*_*xyz*_) for *k* = 0, 2, ⋯, 20 for all 1645 architectures (Figure 2(b)). Strikingly, we found that for each moment order *k*, all architectures lied on the same line, suggesting that all architectures share the same functional form. As a reminder, the function describes the spatial density of roots, which is a probability distribution that specifies, at each point in 3D space, the probability of finding a branch at that point. Deviation from the line increases with higher moment orders because higher moment orders have a much larger range of values (from approximately 10^20^ to 10^100^), and because they capture increasingly fine branching structures. Nonetheless, each line closely approximated the data (*R*^2^ > 0.952 for each of the 11 least-squares regression lines).

For the second test, we plotted the slopes of the lines calculated in the first test versus *k*, the moment order (Figure 2(c)). The slope of this line was 1.020 ± 0.007, meaning that the root architectures deviate by only ≈2% from being truly population-similar. We tested the robustness of this result in three ways. First, we repeated the second test using both even and odd moments between 0 and 20; this yielded a nearly identical slope of 1.021 ± 0.007, suggesting that enough moments are being considered. Second, we computed the slope for all species together, while only sampling 100 random Azucena x Bala plants, instead of using all 1175 of them; we found a similar slope of 1.026 ± 0.009, suggesting that the strong representation of Azucena x Bala plants in our dataset does not unduly bias our results. Third, we computed the slope for each species with at least 100 architectures independently and found some species-specific differences in scaling, as expected, but qualitatively similar results: *Arabidopsis* (1.070 ± 0.022), rice (1.052 ± 0.010), and corn (0.908 ± 0.038).

Notably, we grew tomato plants in “nonoptimal” growth conditions and found significant deviation from population similarity (Figure S1), suggesting that population similarity is not inevitable under every growth regime and may indeed be an actively generated property.

### 2.4. What Is the Functional Form of Root Spatial Densities?

The tests above indicate that all 1645 architectures are population-similar and share a single density function. Next, we seek to find a function with few parameters that gives us a sufficient statistical description of the root spatial density function. For all population-similar functions, the plot of moment order versus slope (Figure 2(c)) will have a slope of 1 [81]. Thus, the only other parameter of the line (the intercept) must provide information about the actual form of the function.

To determine the form of the function, we compared the intercepts from Figure 2(c) to the intercepts of two simple density functions: a 3D uniform function and a 3D Gaussian function truncated at a spherical boundary. A uniform function implies that root density is equal everywhere within the volume occupied by the root system. A Gaussian function implies that root density is highest at the center of mass and then decays as you move outward, with the rate of decay controlled by the standard deviations in each orthogonal direction. The truncation (boundary) of both functions corresponds to the edge of the volume that the root system occupies, outside of which there is zero density.

Strikingly, we found that all the root architectures can be described as a 3D Gaussian truncated at approximately 3.3 standard deviations from the center of mass (Figure 2(d)). We compared the goodness of fit of this function by comparing the intercepts of the root architectures to the intercepts of the uniform function and to a Gaussian function with a higher and lower standard deviation—both of which are poorer fits (Figure 2(d)). Indeed, a difference of only one standard deviation (2.3 or 4.3) significantly departs from the data.

In summary, we derived a simple, statistical description of the spatial density function of all 1645 architectures. A truncated Gaussian density function (Figure 1(b)) implies that only 4 parameters are required to describe statistically how roots are distributed in space: the center of mass and the standard deviations in each of the three spatial growth directions.

### 2.5. Is There Variation in This Property across Species or Genotypes?

Our results suggest a statistical similarity across many diverse architectures, but it is also to be expected that there exists some variation in spatial densities across species or genotypes. Differences in the apparent visual similarities of root architectures can have profoundly different impacts on a plant's growth.

To explore this, we focused on scatter around the regression lines for each moment order to test if this scatter represented noise or systematic differences in architectures from different species or genotypes (Supplementary Methods). We studied scatter around the *k* = 0 line, corresponding to the total mass of the root system (*m*_0_) versus its volume (*σ*_*xyz*_). Recall that this measure of volume is derived entirely from the moments and highly correlates with the convex hull volume (Figure 2(a)). Mass and volume showed a linear relationship on a log-log plot, indicating that as total mass increases, the volumetric space occupied increases according to power law. Thus, roots below the regression line have a smaller total mass for the same volumetric space, and roots above the regression line have a larger total mass for the same volume. Scatter in any of the 11 moment lines can be studied in this manner, but we chose mass and volume because they are relatively easy to interpret.

**Figure 3:**
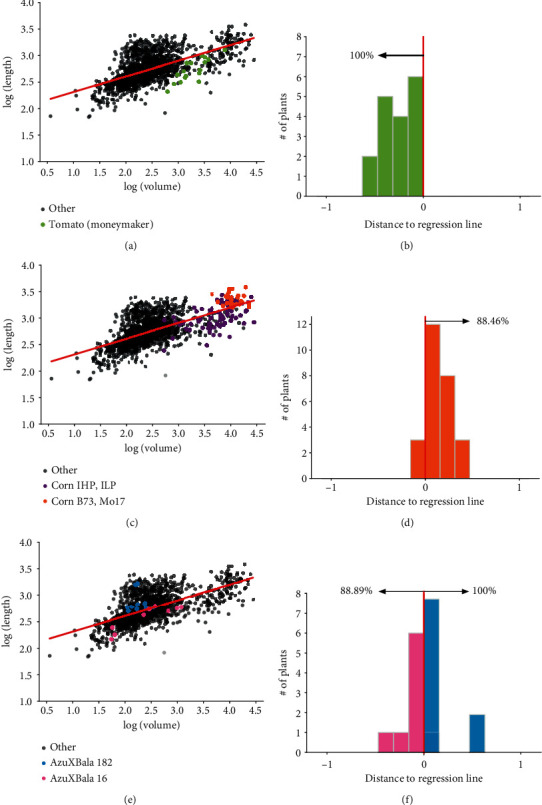
Species- and genotype-specific variation in the spatial density function. (a) Log-log plot of the volume versus mass for all 1645 architectures. Green dots correspond to the tomato WT/Moneymaker architectures, and black transparent dots correspond to all other architectures. (b) Histogram of the distances to the regression line in panel (a) for all tomato WT/Moneymaker architectures; 100.0% of the architectures lie below the regression line. (c, d) Similar plots for four genotypes of corn. (e, f) Similar plots for two recombinant inbred lines of rice (families 182 and 16). Overall, some of the scatter around the regression lines relates to differences in genotype and species.

We found systematic differences in the mass-to-volume relationship across species and across some genotypes within the same species. For example, Figures 3(a) and 3(b) show that 100.0% of tomato WT/Moneymaker plants lay below the regression line, indicating that they had a smaller mass than other root systems, despite occupying the same volume. Figures 3(c) and 3(d) shows that 88.46% of the root systems from the corn B73 and Mo17 genotypes lie above the regression line, whereas the other two corn genotypes (IHP and ILP) lie roughly equally above and below the line. We found similar differences across genotypes of rice, with one genotype (family 182 of Azucena x Bala) laying entirely above the regression line and another genotype (family 16 of Azucena x Bala) laying almost entirely below the regression line (Figures 3(e) and 3(f)).

## 3. Discussion

We studied the spatial density functions of 1645 root systems and found that all architectures were population-similar. Population similarity means that the root density function of all architectures can be transformed into one another by stretching or compressing along orthogonal spatial directions. We also found that a single function, a 3D Gaussian truncated at roughly 3.3 standard deviations, closely describes the spatial densities of the architectures studied. This, as opposed to having different functions for different genotypes or species, was not an expected result. There are clearly other, more complicated functions that may also fit this data, but our goal here was to find a simple function with relatively few parameters that provided a robust fit. Evolutionarily, developing the regulatory circuits to generate and fine-tune a single functional form appears to be parsimonious, and uncovering the molecular mechanisms driving this form is a natural next question.

The density property revealed in this work was identified by a large-scale analysis of entire, nonsimulated, 3D root system architectures across multiple species. Therefore, the growth property incorporates actual heterogeneities in root growth, since it was biologically produced. We pooled data from different species to test if this property may be shared by a diverse set of architectures and thus a useful root phenotyping trait; a per-species analysis, where each species was analyzed separately, also showed similar trends. While all root systems we examined fit under the Gaussian function truncated at approximately 3 s.d., the clustering of species and/or genotypes in groups that deviated from this function is of great interest and highlights potential differences in underlying genetic mechanisms that pattern growth. We consider this a new trait, or phenotype, from which to evaluate root system architecture comprehensively and to constrain architecture modeling schemes.

A previous analysis of 557 shoot architectures—comprising three species (tomato, tobacco, sorghum), each grown in several growth conditions (ambient light, high-light, high-heat, drought, shade), and across roughly 20 developmental time-points per plant—found that the spatial density of shoot architecture branches could also be described by a Gaussian function truncated at approximately 2.0 standard deviations [54], instead of 3.3 standard deviations found here. The truncation amount indicates how many standard deviations away from the center of mass there exists nonzero root density. The higher truncation means that root architectures are more spread-out (relative to their mass) than shoot architectures. There is substantial interest in understanding the growth strategies that root systems use to forage (e.g., [24]), and our results provide a new constraint on this process and perhaps a property common with shoot foraging.

Finally, there are many biological branching structures where Gaussian spatial densities are not observed (e.g., blood vessel networks [6], sand dune morphologies [85, 86], and dendrites of retinal ganglion cells [87]), indicating that these properties should not be considered the null hypothesis. Indeed, given that along with providing support, the main drive for a plant to construct roots is to gather nutrients from the soil, the most obvious design would be space filling (i.e., a uniform spatial density). When an engineer designs anchors for a building, they extend supports equally in a compact space. When a landscape designer spreads irrigation, they do so evenly over the space. Why then have evolutionary processes selected Gaussian distributions in these species, with density concentrated near the center and fewer roots spaced out over an extended region? One possibility is that Gaussian functions balance maximal reach of supporting structures with efficiently gathering resources, e.g., as observed in river systems [88]. Alternatively, the Gaussian densities may simply reflect the competition between roots for space since overlapping Gaussians generate a maximally “flat” combined distribution, as observed in retinal ganglion cells [89]. The well-known central limit theorem that generates Gaussians for random, independent processes does not apply here because the roots are self-avoiding and cannot overlap each other. In such a case, the expected distribution for a random process is strongly non-Gaussian [90]. While the details of the growth process remain to be discovered, our results show that the roots expend resources to array themselves in a specific structure.

Prior work also did not find a similarity between plant-fungus interaction networks below ground and plant-animal interaction networks above ground [91]. Of course, we do not expect every architecture to abide by this rule (e.g., vine-like root systems that do not branch), and it remains to be seen how these properties are affected by changing environmental conditions and across the thousands of other species of plants in the natural world. Nonetheless, this property appears to be relevant to at least 4 species—including two monocots and two dicots, comprising 10 genotypes—and thus may represent a broader principle of plant architecture design.

## Data Availability

Point cloud data for all 1645 3D root architectures are available online at: http://plant3d.navlakhalab.net.

## Supplementary Materials

Supplementary Materials Supplementary Methods. Mathematical definition of population similarity, Mathematical definition of power-law scaling, Example of deviation from population similarity, Analysis of skeleton architectures, Analysis of rotated architectures.
