# Supplementary material for: A Statistical Growth Property of Plant Root Architectures
Source: Plant Phenomics. 2020 Nov 8;2020:2073723. doi: 10.34133/2020/2073723 (PMC7706341; doi:10.34133/2020/2073723)
Supplement: Supplementary Materials — Supplementary Methods. Mathematical definition of population similarity, Mathematical definition of power-law scaling, Example of deviation from population similarity, Analysis of skeleton architectures, Analysis of rotated architectures. [file 2073723.f1.pdf]

A statistical growth property of plant root architectures

Supplementary Information

Sam Sultan*[1], Joseph Snider*[1,2], Adam Conn[1], Mao Li[3], Christopher N. Topp†[3], and Saket Navlakha†[1,4]

[1]The Salk Institute for Biological Studies, Integrative Biology Laboratory
La Jolla, CA USA

[2]University of California San Diego, Institute for Neural Computation, La Jolla, CA

[3]Donald Danforth Plant Science Center, St. Louis, MO

[4]Cold Spring Harbor Laboratory, Simons Center for Quantitative Biology, Cold Spring Harbor, NY

*Co-first authors

†Co-corresponding authors: ctopp@danforthcenter.org, navlakha@cshl.edu

Supplementary Methods

We used a gel-based optical imaging platform to reconstruct 3D architectures of freely-growing root systems [1]. Such a platform provides homogeneous conditions, which allow direct comparisons of one genotype to another, or one species to another. Further, growth conditions such as light, temperature, nutrient and water availability, and bulk density are easy to manipulate and control in the gel system. As advanced soil-based imaging technologies, such as X-ray computed tomography [2–5] and magnetic resonance imaging [6–8] become capable of capturing freely-growing 3D root architectures at high-throughput, our methods can be directly applied.

The root architecture models are generated by visual hull reconstruction via volume carving [9]. Before the reconstruction, an equidistantly spaced voxel grid is defined. Each voxel was projected to a pixel in each 2D image. If the voxel is projected to foreground (root pixels) in all the 2D images, the voxel belongs to the root. The visual hull is the union of all these root voxels. The inner parts of the roots are kept in the visual hull since they are always projected to the foreground regions. The models are thus voxel-based and the resulting .out file used as inputs for this study are volumetric representations (3D coordinates of voxels) of the entire root system, both surface and interior.

Data pre-processing. Each root system architecture was represented as a point cloud with n points, and with each point having an (x, y, z)-coordinate. All root architectures are aligned in their natural (up/down) growth direction. To ensure all root systems were on the same unit scale we normalized each density function by the 0th moment, m_0. No other pre-processing steps were used. All root systems were sampled at 1mm per pixel.

Measure of architecture size using moments. To measure size, we used the standard deviation of the architecture in all directions. This denotes the amount the architecture spreads around the center of mass.

The 2nd moment, m_2, corresponds to the variance; thus, the standard deviation equals:

$$\begin{aligned}\sigma_{xyz} &= \sqrt{\frac{m_2}{m_0}} \\ &= \sqrt{\frac{\sum_i (x_i - \bar{x})^2 (y_i - \bar{y})^2 (z_i - \bar{z})^2}{N}},\end{aligned}$$

where we normalize by the total mass (m_0) to scale to unit mass, and N is the total number of points in the point cloud.

Mass vs. volume comparison in Figure 3. Roots were reconstructed in 3D from scans of 2D images, as described by Topp et al. [1]. This process created a set of voxels representing the 3D architectures, but at variable resolution. While the population-similarity test is specifically designed to be robust to resolution, the representation of total mass is very sensitive to the resolution of the representation. To create a consistent representation across scanning sessions, we skeletonized the voxel representation using PypeTree [10]. From these skeletons, we recalculated all of the moments and found scaling results (1.0595 ± 0.005) that were similar to the more direct measure reported in the main text using the 3D point clouds. To calculate the moments from the skeletonized representation we used an existing code base [11] (available at `http://www.github.com/oldstylejoe/3d_neuron2`) that numerically integrates the moment function along the lines using the GNU Scientific Library integration routines (61 point Gaussian Quadrature, error less than 1e-7). The resulting data were only used to generate the mass and volume measurements in Figure 3.

Mathematical definition of population similarity

Population similarity assumes that all architectures are larger and smaller scaled versions of a reference architecture. In this Supplement, we derive some surprisingly robust consequences of population similarity given the straightforward assumption that small changes in size lead to small changes in shape. To simplify the derivation, we will also assume that there is no preferred direction. These derivations are provided here for convenience, but they are based on standard work [12].

The fundamental equation is to relate arbitrary density functions to scaled versions of a single reference function:

$$f(\mathbf{x};\lambda) = g(\lambda) f_R(\mathbf{x}/\lambda;1), \tag{1}$$

where we choose $\lambda = 1$ as a reference architecture that we stretch (divide by λ) and scale (multiply by $g(\lambda)$). The choice of a reference architecture is arbitrary and depends on conventional choice of units: any of the architectures from a given set of population-similar architectures would serve equally well as a reference. Scaling of the axis by dividing by λ is a coordinate transformation, but the scaling function $g(\lambda)$ requires some investigation.

For example, we might consider a 1D case of a family of line segments of various lengths, centered at 0 and normalized to an area under the curve of 1. Clearly, they all belong to the same population similar family of "box" functions, $f_B(x)$. If we take a reference architecture as a function that is 1/2 on the interval from $[-1, 1]$ and 0 otherwise, then we can write arbitrary segments as:

$$f_B(x;\lambda) = g(\lambda) f_B(x/\lambda;1).$$

Since we require that the area under the curve is always 1 for any λ, we can find g by integrating:

$$\begin{aligned} 1 &= \int dx f_B(x;\lambda) \\ &= \int dx g(\lambda) f_B(x/\lambda;1) \\ &= g(\lambda)\lambda \int d(x/\lambda) f_B(x/\lambda;1) \\ &= \lambda g(\lambda), \end{aligned} \tag{2}$$

or

$$g(\lambda) = \lambda^{-1}.$$

In other words, we have derived that the members of the population similar family of segments centered at 0 with area 1 may be written as

$$f_B(x;\lambda) = \lambda^{-1} f_B(x/\lambda;1),$$

or we can rescale all such segments to look identical by dividing the axis and expanding the height of the segment by a power function of how much we scaled the segment.

Formally, if we assume continuity, i.e., small changes in the length scale result in small changes in the architecture, then very generally we can decide how to normalize our line segments or any arbitrary function so that $g(\lambda) = c\lambda^b$ [12–14], and we recover the well-known power law relationships of nature [15].

In the situation at hand, our goal is to find measurable quantities, given that we lack explicit access to the length scale, λ. One thing we can measure is moments of the architectures that are calculated as sums over position and distance (Equation 1 in the main text). For convenience here, we will represent the sums as integrals. Then the moments may be written as:

$$m_k = c\lambda^{b+3k+3} \int dxdydz x^k y^k z^k f(x,y,z;1), \tag{3}$$

where we plugged in the scaling relation and changed variables. The term under the integral in Equation 3 is entirely in terms of the reference architecture with $\lambda = 1$: it is just a number that contains information about the density function, so we define it to be A_k. Separately, the power law outside the integral represents how the moments vary with changes in length scale (see the next section). Conveniently, when we take ratios of the moments:

$$\begin{aligned} \frac{m_k}{m_0} &= \frac{c\lambda^{b+3k+3} A_k}{c\lambda^{b+3} A_0} \\ &= \frac{A_k}{A_0}\lambda^{3k}. \end{aligned} \tag{4}$$

We reach the explicit goal of removing λ by noting that the ratio of the second and zeroth moments is $m_2/m_0 = A_2/A_0\lambda^6$. Conventionally, we define $\sigma_{xyz} = \sqrt{m_2/m_0}$, and express moment relationships in terms of $\sigma_{xyz} = \sqrt{A_2/A_0}\lambda^3$, leaving:

$$\frac{m_k}{m_0} = \frac{A_k}{A_0}\sqrt{\frac{A_0}{A_2}}\sigma_{xyz}^k,$$

or taking a log of both sides:

$$\log\left(\frac{m_k}{m_0}\right) = k\log\left(\sigma_{xyz}\right) + \log\left(\frac{A_k}{A_0}\sqrt{\frac{A_0}{A_2}}\right). \tag{5}$$

Thus, we recover the linear relationship between moments that underlies the test for population similarity: for each k, the slope of $\log(m_k/m_0)$ versus $\log\sigma_{xyz}$ equals k. Also, we can test if a function, e.g. a Gaussian, is compatible with the data from the second log term that only involves A_k, integrals over the reference function.

We also note that we only measure at finite resolution and approximate the integrals as sums in all cases:

$$m_k \approx \sum_i \Delta_x\Delta_y\Delta_z x_i^k y_i^k z_i^k. \tag{6}$$

In the experimental point clouds, the resolution is the same in all three directions, and we drop the x, y, z subscripts from Δ, or, canceling the Δ's

$$\frac{m_k}{m_0} \approx \frac{1}{N}\sum_i x_i^k y_i^k z_i^k,$$

where $N = \sum_i 1$ is the total number of points in the point cloud.

Finally, in the case that the scaling is different in different directions, the fundamental population similarity equation (Equation 1) may be generalized to use different exponents in the different directions. Instead of dividing each coordinate by λ, we introduce additional scaling exponents, $\beta_{x,y,z}$ in each direction, and divide by $\lambda^{\beta_{x,y,z}}$. However, since we always use ratios of the moments, the exponents cancel out, and there is no effect on the population similarity test.

Note: Population similarity is fundamentally different from the commonly studied notion of image morphing, where any two images can be morphed into one another. In testing for population scaling, we tested if there exists a single family of functions that describes hundreds of samples from a population; i.e., there is a single reference member of the population that all members can scale onto with a single, continuous, low-parameter transformation function. For images, there is clearly no single "reference" image that all possible images can be transformed onto nor a single transformation function.

Mathematical definition of power-law scaling

For convenience, we now sketch the derivation of power laws from the fundamental population similarity equation. This is intended only as an outline, and the interested reader is referred, e.g., to Barenblatt's textbook level treatise [12]. Consider a slightly simplified version with

$$f(\lambda x) = g(\lambda) f(x), \tag{7}$$

which expresses the idea that a scaled version of the function f is identical to f itself. If we allow ourselves to take derivatives in both x and λ, then

$$\begin{aligned} \frac{d}{d\lambda} &\longrightarrow x f'(\lambda x) = g'(\lambda) f(x) \\ \frac{d}{dx} &\longrightarrow \lambda f'(\lambda x) = g(\lambda) f'(x) \end{aligned} \tag{8}$$

We can eliminate the $f'(\lambda x)$ from these two equations and are left with

$$g'(\lambda) = \frac{x g(\lambda) f'(x)}{\lambda f(x)}. \tag{9}$$

Next we use the reference architecture at $\lambda = 1$ as a boundary condition so that

$$\begin{aligned} g'(1) &= \frac{x g(1) f'(x)}{f(x)} \\ &\implies \frac{x f'(x)}{f(x)} = \frac{g'(1)}{g(1)} \equiv b, \end{aligned} \tag{10}$$

where b is a constant.

Plugging back into Equation 9 leaves a differential equation for g alone

$$g'(\lambda) = b \frac{g(\lambda)}{\lambda}, \tag{11}$$

which is the standard d'Alembert equation with the desired power law solution

$$g(\lambda) = c \lambda^b. \tag{12}$$

So, starting with the definition of population similarity leads to power law relationships. Several assumptions here are actually stronger than required, but are in place to move the exposition along. For example, while differentiability with respect to λ is a key assumption, one can weaken the requirement of differentiability with respect to x to generalize to non-smooth $f(x)$.

Example of deviation from population similarity

To investigate whether population similarity is actively generated, as opposed to an emergent constraint, we performed additional experiments with 89 tomato plants, using two genotypes (34 Moneymaker, and 55 from the tomato RNAi line). These plants showed a different genetic-by-environment interaction that resulted in the root tips to curl (Figure S1A). These plants departed significantly from true population-similarity: slope of 1.18 ± 0.04 compared to 1.02 ± 0.007 for the full dataset (Figure S1B–C). This suggests that population-similarity is not inevitable under every growth regime. It is unknown why we observed the curliness, though we speculate that it could be due to changes in ethylene production that occur occasionally when growing plants in conditions without a lot of flow (like a gel).

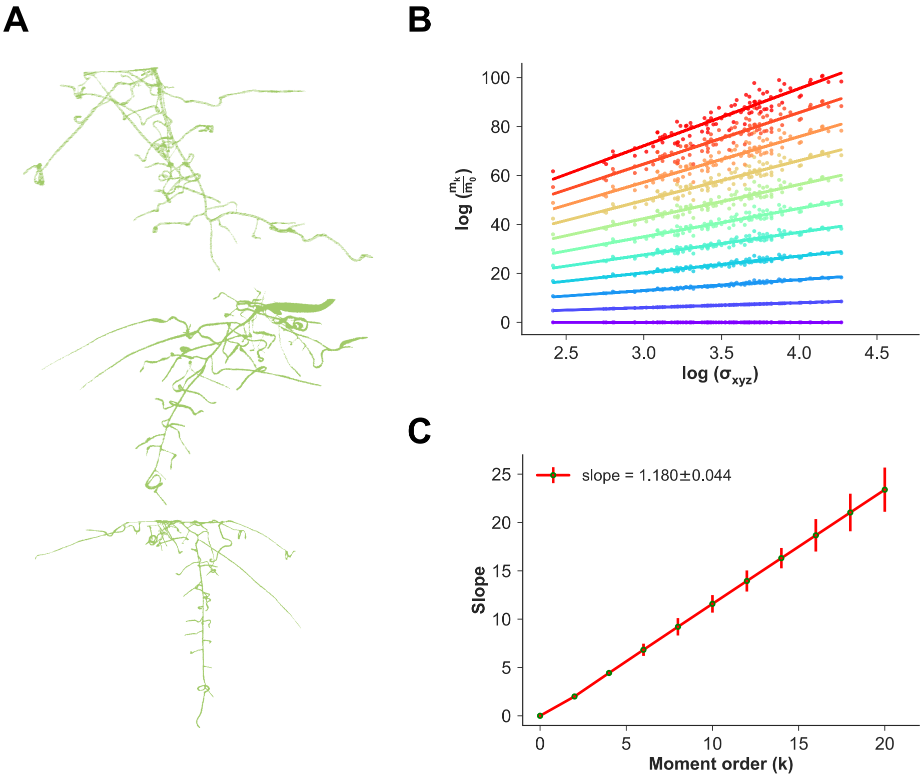

Figure S1: **An example where population-similarity breaks.** A) Three example architectures of "curly" tomato plants. B) Step one of the population similarity test. C) Step two of the test, showing significant deviation from true population similarity; a slope of 1.18 ± 0.04 vs. 1.00 for true population-similarity.

Analysis of skeleton architectures

Prior analyses of spatial distributions used skeletonized versions of architectures [11]. However, the experimental technique used to capture roots generated a grid of equally spaced 3D points. Skeletonizing the data would take us farther from the experimental data; for example, skeletonization loses information about branch thickness and is not as faithful to curvature. Nonetheless, we skeletonized the cloud points using the PypeTree algorithm [10] to fit linear segments to the points and construct graph-theoretic trees. Overall, this coarser analysis lost some fidelity but largely agreed (Figure S2) with our results using 3D point clouds.

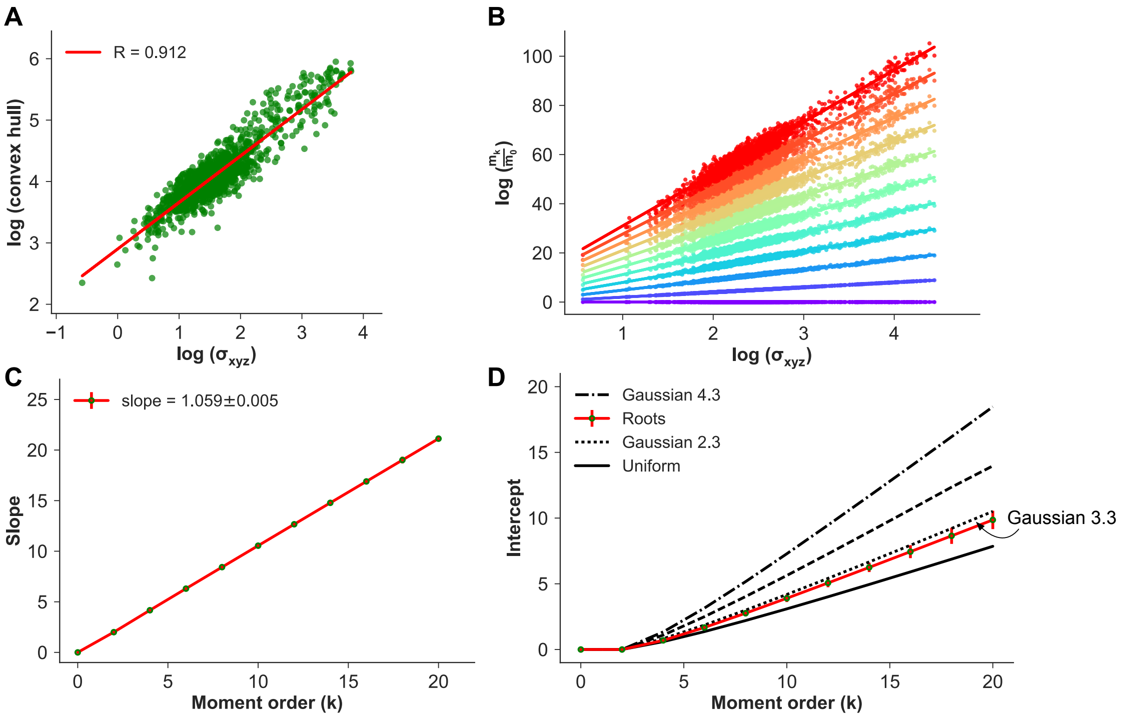

Figure S2: **Analysis of skeletonized architectures reveals similar spatial density properties as analysis using 3D point clouds.**

Analysis of rotated architectures

A Gaussian distribution in 3D space has 3 variance and 3 co-variance components. We can, however, eliminate three of these components by orienting the coordinate system such that the three co-variances (x-y, x-z, and y-z) are all zero. If we perform this rotation, then we still observe population-similarity and a similar cut-off for the truncated Gaussian (Figure S3). The reason we did not report these results in the main text is because we wanted to study the roots in their original orientation, which is their natural growth direction. Finally, the center of the coordinate system aligns with the center of mass, which is at (0,0,0). We can represent the center of mass using only one parameter. Thus, the density function can be specified using four parameters.

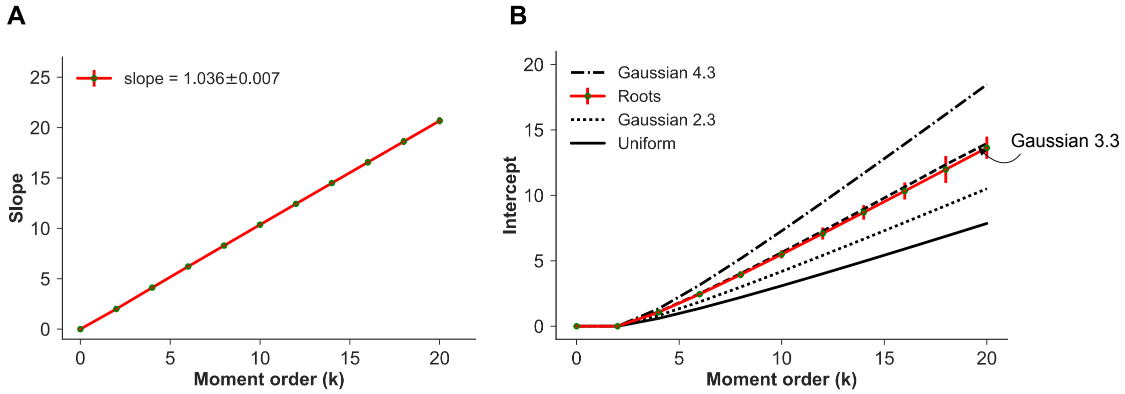

Figure S3: **Rotated architectures have a similar level of population similarity as non-rotated architectures, and have a similar functional form (Gaussian truncated at approximately 3.3 s.d.)**

References

[1] Topp, C. N., Iyer-Pascuzzi, A. S., Anderson, J. T., et al. “3D phenotyping and quantitative trait locus mapping identify core regions of the rice genome controlling root architecture”. *Proc. Natl. Acad. Sci. U.S.A.* 110.18 (2013), E1695–1704.

[2] Mairhofer, S., Zappala, S., Tracy, S., et al. “Recovering complete plant root system architectures from soil via X-ray micro-Computed Tomography”. *Plant Methods* 9 (2013), pp. 1–7.

[3] Kaestner, A., Schneebeli, M., and Graf, F. “Visualizing three-dimensional root networks using computed tomography”. *Geoderma* 136 (2006), pp. 459–469.

[4] Mooney, S., Pridmore, T., Helliwell, J., and Bennett, M. “Developing X-ray computed tomography to non-invasively image 3D root systems architecture in soil”. *Plant Soil* 352 (2012), pp. 1–22.

[5] Tracy, S., Roberts, J., Black, C., et al. “The X-factor: visualizing undisturbed root architecture in soils using X-ray computed tomography”. *J Exp Bot* 61 (2010), pp. 311–313.

[6] Borisjuk, L., Rolletschek, H., and Neuberger, T. “Surveying the plant’s world by magnetic resonance imaging”. *Plant J.* 70.1 (2012), pp. 129–146.

[7] Van as, H. “Intact plant MRIfor the study of cell water relations, membrane permeability, cell-to-cell and long distance water transport”. *J Exp Bot* 58.4 (2007), pp. 743–756.

[8] Dusschoten, D. van, Metzner, R., Kochs, J., et al. “Quantitative 3D Analysis of Plant Roots Growing in Soil Using Magnetic Resonance Imaging”. *Plant Physiol.* 170.3 (2016), pp. 1176–1188.

[9] Ying Zheng, Gu, S., Edelsbrunner, H., et al. “Detailed reconstruction of 3D plant root shape”. *2011 International Conference on Computer Vision.* 2011, pp. 2026–2033.

[10] Delagrange, S., Jauvin, C., and Rochon, P. “PypeTree: a tool for reconstructing tree perennial tissues from point clouds”. *Sensors (Basel)* 14.3 (2014), pp. 4271–4289.

[11] Snider, J., Pillai, A., and Stevens, C. F. "A universal property of axonal and dendritic arbors". *Neuron* 66.1 (2010), pp. 45–56.

[12] Barenblatt, G. *Scaling, Self-similarity, and Intermediate Asymptotics: Dimensional Analysis and Intermediate Asymptotics*. Cambridge Texts in Applied Mathematics. Cambridge University Press, 1996.

[13] Aczel, J. and Dhombres, J. *Functional equations in several variables*. Cambridge University Press (CUP), 1989.

[14] Stevens, C. F. "Darwin and Huxley revisited: the origin of allometry". *J. Biol.* 8.2 (2009), p. 14.

[15] Brown, J. H., Gupta, V. K., Li, B. L., et al. "The fractal nature of nature: power laws, ecological complexity and biodiversity". *Philos. Trans. R. Soc. Lond., B, Biol. Sci.* 357.1421 (2002), pp. 619–626.
